# Supplementary material for: A tentacle for every occasion: comparing the hunting tentacles and sweeper tentacles, used for territorial competition, in the coral Galaxea fascicularis
Source: BMC Genomics. 2020 Aug 8;21:548. doi: 10.1186/s12864-020-06952-w (PMC7430897; doi:10.1186/s12864-020-06952-w)
Supplement: Supplementary file 1 — Additional file 1. Supplementary figures 1 and 2, Supplementary Tables 1-5. [file 12864_2020_6952_MOESM1_ESM.docx]

**Supplementary Information for:**

**A tentacle for every occasion: comparing the hunting tentacles and sweeper tentacles, used for territorial competition, in the coral *Galaxea fascicularis***

Oshra Yosef^1^, Yotam Popovits^1^, Assaf Malik^1, 2^, Maya Ofek-Lalzer^2^, Tali Mass^1^*, Daniel Sher^1^*

^1^ Department of Marine biology, Leon H, Charney school of Marine Sciences; ^2^ Bioinformatics support unit, University of Haifa, Israel

* Corresponding authors: [tmass@univ.haifa.ac.il](mailto:tmass@univ.haifa.ac.il); [dsher@univ.haifa.ac.il](mailto:dsher@univ.haifa.ac.il)


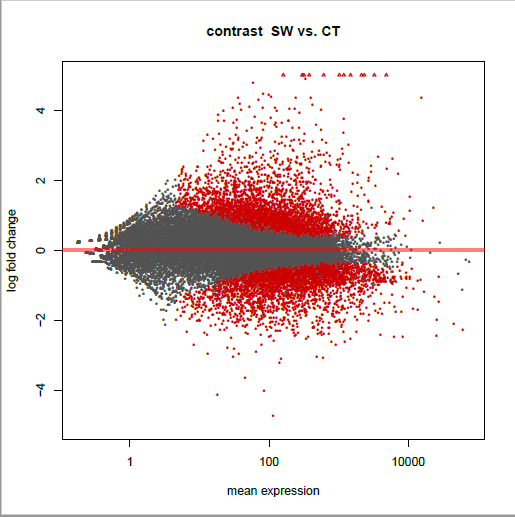


**Supplementary Figure 1:** MA plot of the differential expression analysis comparing the Sweeper and the Catch Tentacles

**
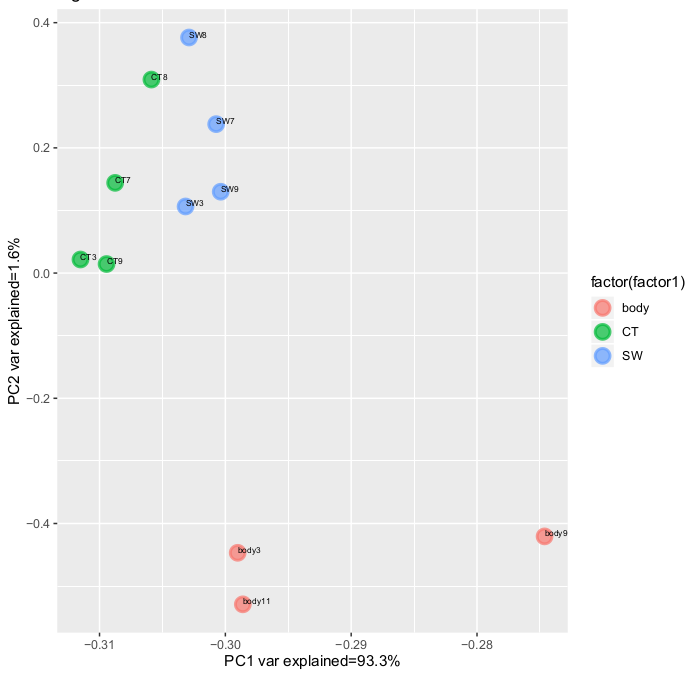
**

**Supplementary Figure 2:** Principle Component representing catch tentacles (green), sweeper tentacles (blue) and whole-body samples (red). Each point represents tentacles collected from a single colony of *G. facsicularies*. Note that two of the three whole-body samples originate from colonies from which catch and sweeper tentacles were not available. Additionally, due to the limited amount of tissue available from the protected *G. fascicularis*, only small parts of the whole-body colonies were sampled, including potentially varying ratios of the body column, the mesenteries and the basal disc. Despite these caveats, the whole-body samples cluster apart from the two tentacle types.

**Supplementary Table 1:** Putative mucin-encoding genes

| **ID** | **Annotation** |
| --- | --- |
| TRINITY_DN99012_c2_g1 | Mucin-like protein (Fragment) OS=Acropora millepora OX=45264 PE=1 SV=1 |
| TRINITY_DN97990_c2_g1 | Mucin-like protein (Fragment) OS=Acropora millepora OX=45264 PE=1 SV=1 |
| TRINITY_DN97616_c0_g3 | Mucin-like protein (Fragment) OS=Acropora millepora OX=45264 PE=1 SV=1 |
| TRINITY_DN97616_c0_g1 | Mucin-like protein (Fragment) OS=Acropora millepora OX=45264 PE=1 SV=1 |
| TRINITY_DN97444_c2_g4 | Mucin-like protein (Fragment) OS=Acropora millepora OX=45264 PE=1 SV=1 |
| TRINITY_DN97444_c2_g2 | Mucin-like protein (Fragment) OS=Acropora millepora OX=45264 PE=1 SV=1 |
| TRINITY_DN97444_c2_g1 | Mucin-like protein (Fragment) OS=Acropora millepora OX=45264 PE=1 SV=1 |
| TRINITY_DN96983_c0_g1 | Mucin-like protein (Fragment) OS=Acropora millepora OX=45264 PE=1 SV=1 |
| TRINITY_DN95678_c1_g12 | Mucin-like protein (Fragment) OS=Acropora millepora OX=45264 PE=1 SV=1 |
| TRINITY_DN95534_c0_g1 | Mucin-like protein (Fragment) OS=Acropora millepora OX=45264 PE=1 SV=1 |
| TRINITY_DN91846_c1_g2 | Mucin-like protein (Fragment) OS=Acropora millepora OX=45264 PE=1 SV=1 |
| TRINITY_DN91846_c1_g1 | Mucin-like protein (Fragment) OS=Acropora millepora OX=45264 PE=1 SV=1 |
| TRINITY_DN87193_c1_g1 | Mucin-like protein (Fragment) OS=Acropora millepora OX=45264 PE=1 SV=1 |
| TRINITY_DN86131_c4_g1 | Mucin-like protein (Fragment) OS=Acropora millepora OX=45264 PE=1 SV=1 |
| TRINITY_DN91846_c1_g2 | Mucin-like protein (Fragment) OS=Acropora millepora OX=45264 PE=1 SV=1 |
| TRINITY_DN105556_c13_g2 | Mucin-like protein (Fragment) OS=Acropora millepora OX=45264 PE=1 SV=1 |
| TRINITY_DN105556_c13_g1 | Mucin-like protein (Fragment) OS=Acropora millepora OX=45264 PE=1 SV=1 |
| TRINITY_DN105389_c5_g1 | Mucin-like protein (Fragment) OS=Acropora millepora OX=45264 PE=1 SV=1 |
| TRINITY_DN103622_c0_g1 | Mucin-like protein (Fragment) OS=Acropora millepora OX=45264 PE=1 SV=1 |
| TRINITY_DN102167_c0_g1 | Mucin-like protein (Fragment) OS=Acropora millepora OX=45264 PE=1 SV=1 |
| TRINITY_DN101562_c0_g1 | Mucin-5AC OS=Homo sapiens OX=9606 GN=MUC5AC PE=1 SV=4 |

**Supplementary Table 2:** Genes encoding putative nematocyst structural proteins

| **ID** | **Annotation** |
| --- | --- |
| TRINITY_DN85790_c0_g1 | Nematogalectin |
| TRINITY_DN92052_c0_g1 | NOWA |

**Supplementary Table 3:** Genes encoding putative GPCRs

| **ID** | **Annotation** |
| --- | --- |
| TRINITY_DN91046_c0_g2 | Histamine H2 receptor |
| TRINITY_DN88700_c1_g1 | Melatonin receptor type 1A |
| TRINITY_DN87578_c3_g2 | Adrenocorticotropic hormone receptor |
| TRINITY_DN87940_c0_g1 | Pyroglutamylated RFamide peptide receptor |
| TRINITY_DN97188_c1_g7 | Neuropeptide FF receptor 2 |
| TRINITY_DN88747_c2_g3 | Follicle-stimulating hormone receptor |
|  |  |
| TRINITY_DN92535_c1_g1 | Beta-1 adrenergic receptor |
| TRINITY_DN15106_c0_g1 | Adenosine receptor A3 |
| TRINITY_DN91294_c0_g2 | Substance-K receptor |
| TRINITY_DN103975_c1_g1 | Histamine H2 receptor |
| TRINITY_DN79884_c0_g1 | Histamine H2 receptor |
| TRINITY_DN91707_c0_g2 | QRFP-like peptide receptor |
| TRINITY_DN91699_c2_g6 | G-protein coupled receptor moody |
| TRINITY_DN94435_c1_g1 | QRFP-like peptide receptor |
| TRINITY_DN95794_c1_g1 | D(1)-like dopamine receptor |
| TRINITY_DN91267_c1_g1 | Orexin receptor type 2 |
| TRINITY_DN90006_c2_g2 | Probable G-protein coupled receptor 83 |
| TRINITY_DN97869_c3_g1 | Substance-K receptor |

**Supplementary Table 4:** Putative toxin-encoding genes

| **ID** | **Annotation** | **TOXIN FAMILY** |
| --- | --- | --- |
| TRINITY_DN101632_c0_g1 | Phospholipase A2 | Phospholipas enzyme family toxins |
| TRINITY_DN97409_c5_g1 | Phospholipase A2 | Phospholipas enzyme family |
| TRINITY_DN97409_c5_g6 | phospholipase A2 Cdr-13 | Phospholipas enzyme family |
| TRINITY_DN93395_c0_g1 | phospholipase A2 | Phospholipas enzyme family |
| TRINITY_DN95538_c2_g9 | phospholipase A2 | Phospholipas enzyme family |
| TRINITY_DN90054_c0_g1 | phospholipase B | Phospholipas enzyme family |
| TRINITY_DN90549_c1_g2 | phospholipase A2 | Phospholipas enzyme family |
| TRINITY_DN88133_c8_g1 | phospholipase D | Phospholipas enzyme family |
| TRINITY_DN99631_c0_g1 | basic phospholipase A2 | Phospholipas enzyme family |
| TRINITY_DN92773_c5_g1 | cytosolic phospholipase A2 | Phospholipas enzyme family cytosolic |
| TRINITY_DN100844_c2_g2 | 85/88 kDa calcium-independent phospholipase A2 | Phospholipas enzyme family |
| TRINITY_DN99947_c1_g1 | cytosolic phospholipase A2 | Phospholipas enzyme family |
| TRINITY_DN101359_c0_g1 | phospholipase A-2 | Phospholipas enzyme family |
| TRINITY_DN90997_c1_g1 | phospholipase B | Phospholipas enzyme family |
| TRINITY_DN105459_c13_g1 | phospholipase B | Phospholipas enzyme family |
| TRINITY_DN89216_c0_g1 | DELTA-alicitoxin-Pse2b | PFT |
| TRINITY_DN86965_c0_g3 | DELTA-alicitoxin-Pse2b | PFT |
| TRINITY_DN153612_c0_g1 | DELTA-actitoxin-Aeq1b | PFT |
| TRINITY_DN94975_c0_g1 | DELTA-alicitoxin-Pse2b | PFT |
| TRINITY_DN97298_c0_g2 | Toxin CrTX-A | PFT |
| TRINITY_DN97962_c4_g1 | Kunitz-type kappaPI-theraphotoxin | Kunitz-type kappaPI-theraphotoxin |
| TRINITY_DN104751_c5_g2 | Zinc metalloproteinase-disintegrin-like halysase | Zinc metalloproteinase-disintegrin-like halysase |
| TRINITY_DN90490_c0_g1 | Astacin-like metalloprotease toxin | Astacin-like metalloprotease toxin |

**Supplementary Table 5:** Putative genes encoding flagella and cilia components

| **ID** | **Annotation** |
| --- | --- |
| TRINITY_DN103322_c2_g2 | 1176097063 ; cilia- and flagella-associated protein 46-like isoform X1 [Orbicella faveolata] ; 48498 ; 2814 |
| TRINITY_DN94545_c0_g3 | 1176108330 ; cilia- and flagella-associated protein 157-like [Orbicella faveolata] ; 48498 ; 557 |
| TRINITY_DN90688_c2_g2 | 1005422771 ; PREDICTED: intraflagellar transport protein 56-like [Acropora digitifera] ; 70779 ; 558 |
| TRINITY_DN97227_c4_g3 | 1176057002 ; cilia- and flagella-associated protein 20 [Orbicella faveolata] ; 48498 ; 193 |
| TRINITY_DN92605_c4_g3 | 1176064189 ; cilia- and flagella-associated protein 77-like [Orbicella faveolata] ; 48498 ; 246 |
| TRINITY_DN94845_c0_g1 | 1005451046 ; PREDICTED: sperm flagellar protein 1-like [Acropora digitifera] ; 70779 ; 367 |
| TRINITY_DN93573_c0_g2 | 1005447406 ; PREDICTED: creatine kinase, flagellar-like [Acropora digitifera] ; 70779 ; 812 |
| TRINITY_DN99122_c2_g1 | 1176110433 ; intraflagellar transport protein 43 homolog A-like isoform X2 [Orbicella faveolata] ; 48498 ; 214 |
| TRINITY_DN99190_c2_g1 | 1176115756 ; intraflagellar transport protein 20 homolog [Orbicella faveolata] ; 48498 ; 129 |
| TRINITY_DN104853_c3_g1 | 1176101238 ; cilia- and flagella-associated protein 161-like [Orbicella faveolata] ; 48498 ; 358 |
| TRINITY_DN104121_c1_g1 | 1176100962 ; intraflagellar transport protein 140 homolog [Orbicella faveolata] ; 48498 ; 1648 |
| TRINITY_DN105559_c3_g2 | 1176112090 ; dynein beta chain, flagellar outer arm-like isoform X2 [Orbicella faveolata] ; 48498 ; 5226 |
| TRINITY_DN105503_c4_g1 | 1176061771 ; intraflagellar transport protein 52 homolog [Orbicella faveolata] ; 48498 ; 452 |
| TRINITY_DN104551_c6_g3 | 1176077915 ; intraflagellar transport protein 22 homolog isoform X3 [Orbicella faveolata] ; 48498 ; 321 |
| TRINITY_DN104787_c2_g2 | 1005454189 ; PREDICTED: cilia- and flagella-associated protein 47-like [Acropora digitifera] ; 70779 ; 2476 |
| TRINITY_DN100702_c6_g1 | 1005462828 ; PREDICTED: cilia- and flagella-associated protein 99-like [Acropora digitifera] ; 70779 ; 619 |
| TRINITY_DN95304_c3_g1 | 1176092471 ; cilia- and flagella-associated protein 43-like [Orbicella faveolata] ; 48498 ; 1972 |
| TRINITY_DN102047_c2_g1 | 1005419733 ; PREDICTED: cilia- and flagella-associated protein 44-like isoform X1 [Acropora digitifera] ; 70779 ; 1918 |
| TRINITY_DN97263_c1_g1 | 1126198640 ; PREDICTED: intraflagellar transport protein 74 homolog isoform X1 [Branchiostoma belcheri] ; 7741 ; 599 |
| TRINITY_DN103525_c1_g1 | 1191034778 ; intraflagellar transport protein 172 homolog isoform X1 [Exaiptasia pallida] ; 1720309 ; 1750 |
| TRINITY_DN98787_c3_g2 | 1176097063 ; cilia- and flagella-associated protein 46-like isoform X1 [Orbicella faveolata] ; 48498 ; 2814 |

**Supplementary Data File 1**: Interactive version of the heatmap in Figure 3B. Clicking on the row will show the gene name, which can then be looked up in the supplementary excel file. This file is supplied as an html file

**Supplementary Data File 2:** Excel table with the transcripts assembled by Trinity, their expression levels and annotations.
